# Thyroid storm with acute liver failure and disseminated intravascular coagulation- lessons in diagnosis and treatment

**DOI:** 10.1186/s40842-024-00182-9

**Published:** 2024-08-24

**Authors:** Manudi Vidanapathirana, Dilushi Wijayaratne

**Affiliations:** 1 National Hospital of Sri Lanka, Colombo, Sri Lanka; 2 Faculty of Medicine, University of Colombo, Colombo, Sri Lanka

**Keywords:** Thyroid storm, Acute liver failure, Disseminated intravascular coagulation

## Abstract

Thyroid storm is a medical emergency with a high mortality rate. Acute liver failure (ALF) and disseminated intravascular coagulation (DIC) are rarely reported with thyroid storm, and their occurrence is unrelated to the degree of free circulating thyroxine.

We present the case of a 41-year-old Sri Lankan female, with a fatal case of thyroid storm. She initially presented with palpitations and heat intolerance, and subsequently developed acute liver failure with hepatic encephalopathy and coagulopathy. There was hypoglycemia and resistant lactic acidosis consequent to the liver failure. The clinical course progressed to DIC and she eventually succumbed to the illness. Treatment comprised the standard management of thyroid storm.

This case report highlights the importance of bearing ALF and DIC in mind as complications of thyroid storm, outlines their pathophysiology, and uses pathophysiological mechanisms to justify, evolving extracorporeal therapeutic strategies for resistant cases.

## Introduction

Thyroid storm is a medical emergency with a high mortality rate of 10–30% [1]. It is characterized by multi-organ involvement in the presence of thyrotoxicosis [2]. The symptom profile of thyrotoxicosis includes fever, palpitations, agitation/psychosis and diarrhea [2]. Life-threatening complications of thyroid storm are congestive cardiac failure, hypo/hypertension, arrhythmias, acute liver failure (ALF) and disseminated intravascular coagulation (DIC) [2].

Interestingly, the pathophysiological basis for the multi-organ involvement in thyroid storm is unrelated to the circulating level of free thyroxine hormone [3]. Rather, it is related to the overproduction of inflammatory cytokines and immunological mediators, occurring in thyrotoxicosis [3]. This overproduction is due to increased sympathetic activity, along with an increased cellular response to thyroid hormones [3].

Management of thyroid storm is with thionamides, beta blockade, iodinated therapy, steroids, bile acid sequestrants and other supportive measures [1]. However, extracorporeal modalities are emerging as newly recognized therapies that may have benefit in resistant cases [4].

ALF and DIC are rare, life-threatening complications of thyroid storm, and this case report will discuss pathophysiological mechanisms and therapeutic strategies of both.

## Case presentation

We present the case of 41-year-old Sri Lankan female who was managed for thyroid storm, with subsequent development of ALF with resistant lactic acidosis and DIC.

This patient presented with recent enlargement of a goiter, which has not been evaluated prior to the current presentation. The progressive enlargement was noted without any pain, for 2 weeks, prior to presentation. She experienced symptoms of thyrotoxicosis such as heat intolerance, weight loss and palpitations. There were features of Graves’ disease such as a smooth, firm, non-tender goiter, proptosis and thyroid bruit. Her temperature was 37.5 degrees Celsius, and she had a pulse rate of 112 beats per minute, with a blood pressure of 150/90 mmHg. Her respiratory rate was 20 cycles per minute with a saturation of 99% on room air. Her Burch Wartofsky score upon presentation amounted to 25, suggestive of an impending thyroid storm [5]. According to Burch Wartofsky point scale, 5 points were given for a temperature of 37.5 degrees Celsius, 10 points for mild agitation and 10 points for a heart rate between 110 and 119 beats per minute (5). Her thyroid stimulating hormone (TSH) level was < 0.0025 mU/L (0.4-4 mU/L) and T4 level was 3.35 ng/dL (0.8–1.8 ng/dL). An ultrasound scan of the neck revealed a diffusely enlarged goiter with increased vascularity, and no nodules were noted.

Within 2 h of presentation, she was started on propanalol 40 mg thrice daily and carbimazole 20 mg thrice daily.

24 h after admission, she developed pyrexia with a temperature of 39 degrees Celsius, worsening palpitations, dyspnea and drowsiness. The palpitations were due to atrial flutter with a heart rate of 144 beats per minute. There was no evidence of hypotension, myocardial ischemia or heart failure. Her blood pressure was 140/70 mmHg, respiratory rate 30 cycles per minute and saturation on room air was 98%. Arterial blood gas (ABG) showed severe high anion gap metabolic acidosis with a pH of 7.08, PaO2 of 97 mmHg, PaCO2 of 31.8 mmHg, lactate of 8.6 mmol/L, bicarbonate of 9.5 mEq/L and a base excess of -18 mmol/l.

The dyspnea in the presence of clear lungs, was attributed to metabolic acidosis with hyperlactatemia. The pH was 7.08, and the lactate level was 8.6 mmol/L. Hyperlactatemia is seen in thyrotoxicosis due to relative hepatic ischemia in the presence of a high metabolic rate. Drowsiness was due to level 3 hypoglycemia with a capillary blood sugar value of 48 mg/dL. On the second day of admission, rising liver enzymes were noted, and it became apparent that the lactic acidosis and the hypoglycemia, were contributed by the presence of acute liver injury. Alternate causes for acute liver injury such as viral hepatitis, autoimmune hepatitis and drugs or toxins were considered. The patient has not taken any drugs or herbal supplements prior to presentation, viral serology for viral hepatitis A and B were negative, and antinuclear antibody level too was negative.

The hypoglycemia was managed with intravenous dextrose, and the arrhythmia was managed with rate controlling beta blockade. Propanolol 40 mg thrice daily was used, and the best control achieved was a heart rate of between 90 and 100 beats per minute. Hydrocortisone 100 mg intravenously, 8 hourly, and cholestyramine 4 g orally, 6 hourly, were administered in accordance with standard management guidelines for thyroid storm. Lugol’s iodine was started on day 3 of hospital stay. Intravenous cefotaxime was empirically started to cover for sepsis while awaiting the results of the septic screen, since infection is a common precipitant of thyroid storm and hyperlactatemia could have been due to infection. However, the septic screen was negative, and inflammatory markers such as C reactive protein, were not suggestive of sepsis.

On the 2nd day of admission, she developed hypotension and required inotropic support, and beta blockers had to be withdrawn. The hypotension was attributed to the arrhythmia and myocardial depression due to lactic acidosis. Mechanical cardioversion reset the rhythm to sinus rhythm. The metabolic acidosis was attempted to be treated with judicious intravenous hydration, intravenous sodium bicarbonate, and in the absence of a response, with haemodialysis (HD). Sustained low-efficiency dialysis (SLED) was done on the 2nd, 3rd and 4th day after admission. On the 3rd day after admission, the disease evolution became detrimental, with development of ALF with both coagulopathy and hepatic encephalopathy. DIC ensued with upper gastrointestinal bleeding.

Tables 1 and 2 show the arterial blood gas and investigation trends, respectively.

She was intubated on the 3rd day of admission due to reduced level of consciousness, attributed to hepatic encephalopathy.

N-acetyl cysteine infusion was commenced for acute liver injury, and fresh frozen plasma infused for correction of coagulopathy contributing to gastrointestinal bleeding. On day 5 of admission, she succumbed to the illness.

**Table 1:** Arterial blood gas trends

ABG parameter	Normal range	Day 2	Day 2	Day 2 (After IV NaHCO3)	Day 3 (Post-HD)	Day 4 (Post-HD)	Day 5
pH	7.35–7.45	7.080	6.9	7.36	7.376	7.41	7.280
pCO2 (mmHg)	35–45	31.8	25	20.8	39.6	45	60
Bicarbonate (mEq/L)	22–29	9.5	4.5	12	23.4	34.8	28.6
Lactate (mmol/L)	< 1	8.6	10	17.5	7.1	5.3	11.4
pO2 (mmHg)	75–100	97	93	87	91	95	85
Glucose (mg/dL)	70–100	124	111	214	343	105	
Base excess (mmol/L)	(-2)-(+ 2)	-18	-20				

pCO2- Partial pressure of carbon dioxide; pO2- Partial pressure of oxygen

**Table 2:** Investigation trends

Investigation	Nornal ranges	Day 1	Day 2	Day 2	Day 3	Day 4	Day 5
WBC (x1000/L)	4–10	4.9	5.27	21.87	31.82	26.14	17.14
Hb (g/dL)	12–15	7.6	7.4	7.6	6.2	7.2 (post transfusion)	7.8
Platelets (x 1000/L)	150–450	148	145	63	54	55	42
CRP (mg/dL)	< 6		10		24		
Na (mmol/L)	135–145	140	141		135	137	138
K (mmol/L)	3.5-5	3.8	3.8		3.7	3.9	4.1
Cr (mg/dL)	0.7–1.2	0.5	0.43		0.5	0.8	1.21
AST (U/L)	8–33	28	29		1410	978	643
ALT (U/L)	4–36	42	41		381	414	316
Albumin (g/dL)	3.4–5.4		3.4				
Total bilirubin (mg/dL)	0.1–1.2	1.9	2.7		6.1	6.3	7.7
Direct bilirubin (mg/dL)	< 0.3	1.0	1.5		3.8	3.8	4.1
Calcium(mg/dl)	8.5–10.2		8.2				
PT (s)	11-13.5		36.6		53.7	67.3	
INR	1		2.82		4.25	5.4	
APTT (s)	21–35		40		45.7	46	
Blood picture					Evidence of microangiopathic haemolytic anaemia		
2D echo					Ejection fraction-55% No regional wall motion abnormalities		
Ultrasound abdomen					Normal liver architecture		

ALT- Alanine transaminase; APTT- Activated partial thromboplastin time; AST- Aspartate transaminase; Cr- Serum creatinine; CRP- C Reactive Protein, Hb- Haemoglobin; INR- International normalized ratio; K- serum potassium; Na- Serum sodium; PT- Prothrombin time; WBC- White blood cell count

## Discussion

The discussion will highlight the importance of recognizing ALF and DIC as possible complications of thyroid storm, delineate the mechanisms of the above complications and propose therapeutic strategies that may improve patient survival.

In terms of liver injury, there is no direct correlation between the degree of thyroid dysfunction and occurrence of liver derangement [3]. The predisposing factors to hepatic dysfunction in thyrotoxicosis also remain poorly understood [3]. Hypothesized mechanisms for ALF are increased T3 causing hepatocyte apoptosis through a mitochondrial-mediated pathway involving caspase 3 and 9, increased T3 causing accumulation of bilirubin precursors due to lack of regulation of bilirubin metabolism, right heart failure with congestive hepatopathy, relative hepatic ischemia due to peripheral vasodilation and increased oxygen demand due to high metabolic activity, and direct injury to hepatocytes due to inability to metabolize excessive levels of thyroid hormones [2, 3].

In our patient’s case, we believe that some, if not all of these may have played a role. The risk factors for ischemia she had were the presence of an arrhythmia, hypotension and the initial presence of high output heart failure. The high output heart failure was evidenced in her case by the presence of wide pulse pressure, warm peripheries and preserved ejection fraction on echocardiography [6]. The wide pulse pressure and warm peripheries indicate significant peripheral vasodilation [6]. We believe her heart failure was more of a forward failure due to vasodilation and high metabolic demand, rather than a backward failure with congestive hepatopathy, due to the absence of elevated jugular venous pulsation and pulmonary venous congestion.

Liver injury itself can lead to DIC through decreased levels of fibrinogen, plasminogen and vitamin K-dependent coagulation factors [2]. Mechanisms unrelated to liver injury also play a part. Thyroid storm is known to cause exaggerated release of pro-inflammatory cytokines and leads to systemic inflammatory response syndrome (SIRS) [2, 3]. Thyroxine, induces the production of interleukin-1 (IL-1). IL-1 causes platelet activation, and increases the production of tissue factor, von Willebrand factor, and interleukins 6 and 8 which generate a pro-thrombotic environment [2].

**Fig. 1:**
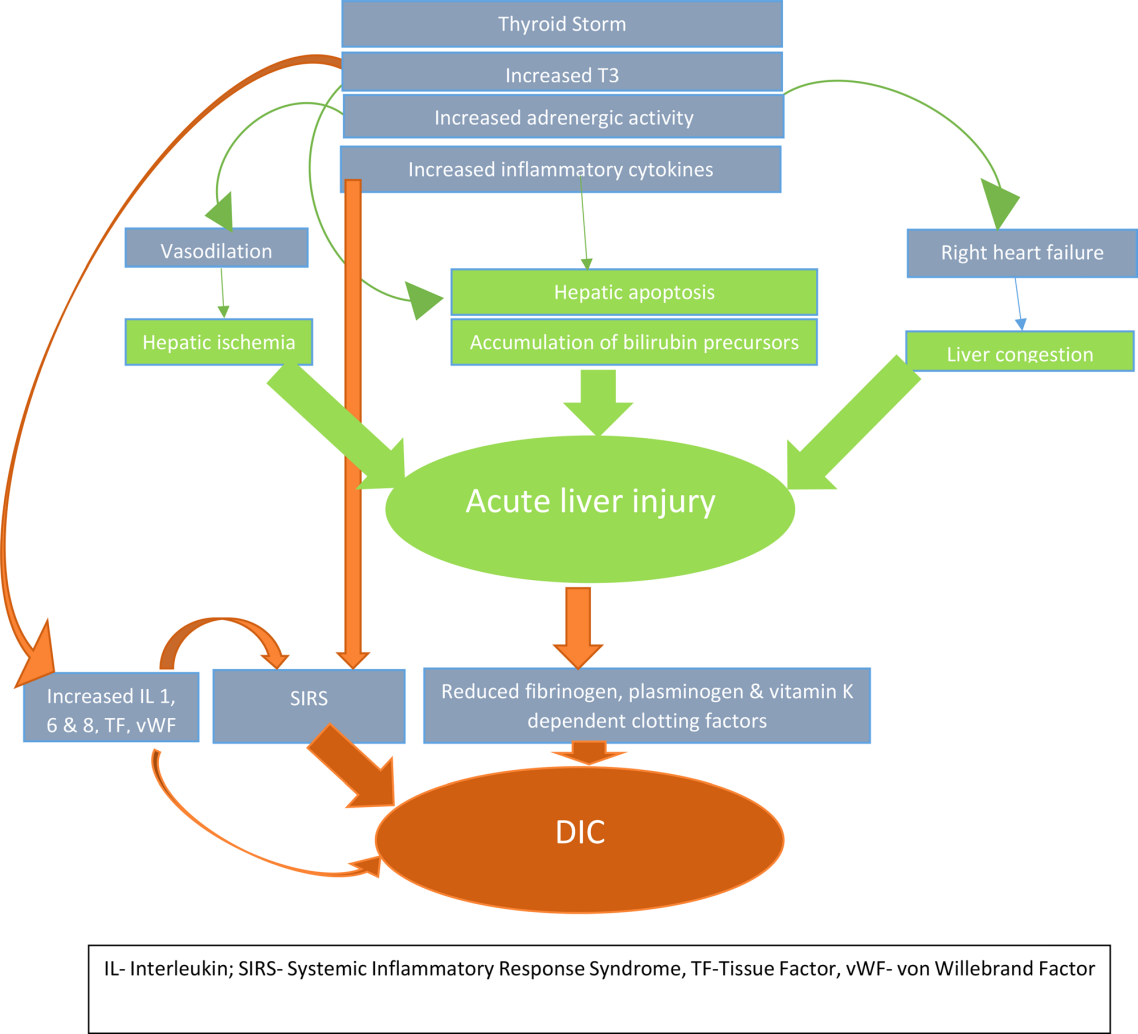
Pathophysiological mechanisms leading to acute liver failure and disseminated intravascular coagulation in thyroid storm

Figure 1 shows the pathophysiological mechanisms that may have played a role in the exponentially detrimental path that our patient’s clinical condition followed.

The resistant lactic acidosis that was seen in our patient was primarily attributed to be due to ALF [7]. However, other mechanisms may also have been at play. Decreased cardiac output due to arrhythmia and increased basal metabolic rate with increased oxygen utilization may have contributed to type A lactic acidosis in this patient [7]. Sepsis as a contributor to lactic acidosis seemed clinically unlikely, and the inflammatory markers and negative cultures also pointed away from it. Vitamin B1 or thiamine deficiency may have also contributed to lactic acidosis in this Sri Lankan female, and due to the unavailability of formal testing, IV thiamine was empirically given as treatment [8].

There were multiple therapeutic challenges in this patient. The first therapeutic challenge was that the degree and rapidity of ALF, SIRS and DIC outpaced the rapidity of onset of action of the standard treatments for thyroid storm. The second therapeutic challenge was that the use of propylthiouracil, which is the preferred thionamide in thyroid storm, also carries with it an inherent risk of liver injury [9]. Although, this usually sets in weeks after the initiation, a few case reports outline cases where the liver injury had set in a few days after initiation [9]. Formularies are ambiguous about the prudence of continuing propylthiouracil in instances of ALI due to aetiologies unrelated to the drug [10]. The proposed mechanism of ALI with propylthiouracil is an immunological reaction to a metabolite [10]. It must therefore, be considered whether the continuation of propylthiouracil in a liver that was already being subjected to multifactorial derangement, added insult to injury in our patient.

The third therapeutic challenge was resource limitation. Extracorporeal measures such as continuous renal replacement therapy (CRRT) and therapeutic plasma exchange (TPE) show evolving promise in the management of thyroid storm and liver failure [11, 12]. Various case reports and case series have shown benefits of these therapies [4, 11, 12].

During TPE, thyroid hormone-binding globulins along with bound thyroid hormones are removed from the circulation and replaced with a colloid such as albumin and/ or fresh frozen plasma [11]. The colloid provides a new binding site for free thyroxine, reducing the amount of circulating free hormone [11]. TPE also helps to remove thyroid receptor stimulating antibodies in Graves’ disease [11]. Other proposed mechanisms through which TPE improves survival in thyroid storm are, reducing the levels of protein-bound thyroid hormones (both T4 and T3), reducing the levels of pro-inflammatory and pro-thrombotic cytokines and removal of 5-monodeiodinase which converts T4 to the active form T3 [12]. Several studies have shown similar effects with CRRT, with some studies showing more of a sustained benefit with CRRT than with TPE [13, 14]. Intermittent HD has not been tried with thyroid storm, possibly since these patients are generally too critically ill to tolerate it.

This patient had 3 indications for use of TPE i.e. thyroid storm, ALF and lactic acidosis. Unfortunately, TPE was not available in our setting, due to resource limitations. It is also important to note that the use of extracorporeal systems serves only as a temporizing measure until the definitive therapies for thyroid storm i.e. thionamides or thyroidectomy come in to action [12].

Our patient, was treated with HD as opposed to CRRT, again due to lack of facilities. The sustained removal of thyroid hormones and slow restoration of the metabolic milieu expected with CRRT is quite possibly, difficult to achieve with HD [4]. It is also possible that HD, caused further haemodynamic fluctuations, worsening liver injury, acidosis and myocardial dysfunction.

There are three reported cases of thyroid storm with ALF successfully treated with thyroidectomy, followed by liver transplantation [15, 16]. In one of them, the period till thyroidectomy was managed with TPE, but in the other two, no extracorporeal modalities had been utilized.

## Conclusions

ALF and DIC are two rare complications of thyroid storm. It is important to be vigilant of these due to their high mortality rates. Understanding the underlying pathophysiology of ALF and DIC in thyroid storm will help to guide possible life-saving treatments.

## Data Availability

All data generated or analyzed during this study are included in this published article.

## Abbreviations

ABG: Arterial blood gas
ALF: Acute liver failure
ALI: Acute liver injury
CRRT: Continuous renal replacement therapy
DIC: Disseminated intravascular coagulation
HD: Haemodialysis
IL: Interleukin
SIRS: Systemic inflammatory response syndrome
SLED: Sustained low efficiency dialysis
TPE: Therapeutic plasma exchange
TSH: Thyroid stimulating hormone

## References

[1] Chiha M, Samarasinghe S, Kabaker AS. Thyroid storm: an updated review. J Intensive Care Med. 2015;30(3):131–40. 10.1177/0885066613498053. 23920160 10.1177/0885066613498053

[2] Kim Y, Gurung D, Sumbly V, Reich DM, Bashir T. Thyroid storm-induced acute liver dysfunction and disseminated intravascular coagulation. Cureus. 2021;13(7):e16504. 10.7759/cureus.16504. PMID: 34430118; PMCID: PMC8375274. 34430118 10.7759/cureus.16504 PMC8375274

[3] Sanna Fatima R, Puri S, Patnaik J, Mora, When. A toxic thyroid makes the liver toxic: a case of thyroid storm complicated by acute liver failure. AACE Clinical Case Reports. 2017;3(3):e200-e204. ISSN 2376 – 0605. 10.4158/EP161496.CR.

[4] Lim SL, Wang K, Lui PL, Ramanathan K, Yang SP. Crash landing of thyroid storm: a case report and review of the role of extra-corporeal systems. Front Endocrinol. 2021;12:725559. 10.3389/fendo.2021.725559. 10.3389/fendo.2021.725559 PMC8417732 34489870

[5] Burch HB, Wartofsky L. Life-threatening thyrotoxicosis. Thyrotoxic storm. Endocrinol Metab Clin North Am. 1993;22(2):263–77. 8325286 10.1016/S0889-8529(18)30165-8

[6] Gregory M, Taylor AMC, Pop EL, McDowell. High-output congestive heart failure: a potentially deadly complication of thyroid storm. Oxford Medical Case Reports. 2019;2019(6):omz045. 10.1093/omcr/omz045. 10.1093/omcr/omz045 PMC6568196 31218012

[7] Prosser JS, Quan DK. Trauma triggering thyrotoxic crisis with lactic acidosis. J Emerg Trauma Shock. 2015 Oct-Dec;8(4):232–4. 10.4103/0974-2700.161656. PMID: 26604530; PMCID: PMC4626941. 10.4103/0974-2700.161656 PMC4626941 26604530

[8] Yoshino T, Kawano D, Azuhata T, Kuwana T, Kogawa R, Sakurai A, Tanjoh K, Yanagawa T. A patient with Graves’ disease who survived despite developing thyroid storm and lactic acidosis. Ups J Med Sci. 2010;115(4):282–6. Epub 2010 Aug 23. PMID: 20731531; PMCID: PMC2971487. 20731531 10.3109/03009734.2010.486908 PMC2971487

[9] Ba JH, Wu BQ, Wang YH, Shi YF. Therapeutic plasma exchange and continuous renal replacement therapy for severe hyperthyroidism and multi-organ failure: a case report. World J Clin Cases. 2019;7(4):500–7. 10.12998/wjcc.v7.i4.500. PMID: 30842962; PMCID: PMC6397818. 30842962 10.12998/wjcc.v7.i4.500 PMC6397818

[10] Williams KV, Nayak S, Becker D, Reyes J, Burmeister LA. Fifty years of experience with propylthiouracil-associated hepatotoxicity: what have we learned? J Clin Endocrinol Metab. 1997;82:1727–33. 9177371 10.1210/jcem.82.6.4011

[11] Koh H, Kaushik M, Loh JK, Chng CL. (2019). Plasma exchange and early thyroidectomy in thyroid storm requiring extracorporeal membrane oxygenation. Endocrinology, Diabetes & Metabolism Case Reports. 2019:19–0051. Retrieved Jan 23, 2024, from 10.1530/EDM-19-0051. 10.1530/EDM-19-0051 PMC6685092 31352696

[12] Muller C, Perrin P, Faller B, Richter S, Chantrel F. Role of plasma exchange in the thyroid storm. Ther Apher Dial. 2011;15(6):522 – 31. 10.1111/j.1744-9987.2011.01003.x. PMID: 22107688. 10.1111/j.1744-9987.2011.01003.x 22107688

[13] Parikh T, Sharma M, Hegde A. SUN-559 refractory thyroid storm: role of single pass Albumin dialysis (SPAD). J Endocr Soc. 2019;3(Suppl 1):SUN–559. 10.1210/js.2019-SUN-559. 10.1210/js.2019-SUN-559

[14] Koball S, Hickstein H, Gloger M, Hinz M, Henschel J, Stange J, et al. Treatment of thyrotoxic crisis with plasmapheresis and single pass Albumin dialysis: a case report. Artif Organs. 2010;34(2):E55–58. 10.1111/j.1525-1594.2009.00924.x. 20420590 10.1111/j.1525-1594.2009.00924.x

[15] Nagakawa K, Soyama A, Hara T, Matsushima H, Imamura H, Tanaka T, Morita M, Kuba S, Adachi T, Hidaka M, Miyaaki H, Akazawa S, Horie I, Sekino M, Hara T, Okano S, Nakao K, Eguchi S. Living donor liver transplantation for a patient with acute liver failure following thyroid storm: a case report. Surg Case Rep. 2023;9(1):208. 10.1186/s40792-023-01786-6. PMID: 38036922; PMCID: PMC10689690. 38036922 10.1186/s40792-023-01786-6 PMC10689690

[16] Hambleton C, Buell J, Saggi B, Balart L, Shores N. J., Kandil E. Thyroid storm complicated by fulminant hepatic failure: Case Report and Literature Review. Annals Otology Rhinology Laryngology. 2013;122(11):679–82. 10.1177/000348941312201103. 10.1177/000348941312201103 24358627

